# Development of a Virtual Home-Based Cycling Intervention for Individuals With Chronic Obstructive Pulmonary Disease: Qualitative Study

**DOI:** 10.2196/71234

**Published:** 2025-11-14

**Authors:** Kristina Krogh Christensen, Henrik Bøggild, Ulla Møller Weinreich, Anna Lei Stoustrup

**Affiliations:** 1 Department of Respiratory Diseases, Aalborg University Hospital Aalborg Denmark; 2 Department of Health Science and Technology, Public Health and Epidemiology, Aalborg University Aalborg Denmark; 3 Department of Clinical Medicine, The Faculty of Medicine, Aalborg University Aalborg, null Denmark

**Keywords:** chronic obstructive pulmonary disease, COPD, development, home-based exercise intervention, virtual cycling exercise, pulmonary rehabilitation

## Abstract

**Background:**

Chronic obstructive pulmonary disease (COPD) is a leading cause of mortality, and exercise has been shown to reduce both. Health conditions, environmental factors, and logistical challenges are often barriers for participation in pulmonary rehabilitation (PR). Given the barriers many individuals with COPD face when attending health care centers for PR, virtual home-based cycling exercise could be an option.

**Objective:**

This study aimed to explore the development of a home-based cycling exercise intervention for individuals with COPD, focusing on aspects such as bicycle selection, app functionality, and pilot testing. Furthermore, it aimed to explore participants' and nonparticipants' attitudes toward the intervention.

**Methods:**

Using a phenomenological-hermeneutic approach, data were gathered from 15 semistructured interviews, including test pilots, participants, and nonparticipants. A thematic analysis was used to analyze the data.

**Results:**

Thematic analysis identified 8 key themes: bicycle selection, individual guidance needs, geographical and video quality, online connectivity, comfort and accessibility of home-based cycling, flexibility, energy levels, and practical limitations. Findings highlighted a preference for pedal bicycles with adjustable intensity, the importance of flexibility in scheduling, and the autonomy provided by a home-based setup. While participants appreciated the virtual journey on videos, barriers such as lack of energy, stress, and limited space were reported by nonparticipants.

**Conclusions:**

Recommendations include enhancing app features and addressing individual needs to improve adherence. The study underscores the potential of tailored home-based exercise interventions in overcoming traditional PR challenges.

**International Registered Report Identifier (IRRID):**

RR2-10.1136/bmjresp-2024-002573

## Introduction

### Chronic Obstructive Pulmonary Disease

Chronic obstructive pulmonary disease (COPD) is the most common lung disease worldwide, affecting over 292 million people, and ranking as the third leading cause of death globally [1]. Admissions due to exacerbations of COPD are frequent, and readmissions are concerning, as a large group of individuals experience recurrent hospitalizations [2].

Despite the known benefits of pulmonary rehabilitation (PR) [3-5], many individuals with COPD experience barriers to participation, such as shortness of breath, comorbidities, weather conditions, logistical difficulties related to transportation and parking, and other physical health issues, significantly limiting the individual’s ability to engage in physical activity [6,7]. To address motivational aspects beyond logistical barriers, self-determination theory (SDT) offers a valuable framework. SDT emphasizes autonomy, competence, and relatedness—three psychological needs that can guide the design of more engaging and sustainable home-based exercise interventions.

To optimize PR programs, it is important to adjust duration and intensity to each individual’s exercise tolerance. This individualized approach ensures that the individual’s specific needs and limitations are addressed, enhancing their ability to participate in physical activity [8,9]. Given these physical limitations and logistical difficulties, there is a need for a more adapted and practical solution to improving participation in PR among individuals with COPD.

### Home Exercise and Cycling Exercise

Given the barriers many individuals with COPD face when attending health care centers for PR, home exercise could be an option. Exercise activities can safely be carried out at home, and among those, cycling can be offered as an alternative. Cycling has been identified as an effective way to improve physical function and is widely accessible [10]. In Denmark, cycling is one of the most preferred forms of transportation and is deeply ingrained in Danish culture [11], with 70% of Danes aged over 16 years owning a bicycle [12]. Cycling accounts for 21% of all trips under 10 kilometers and 15% of all transportation in the country [11].

### 4Mvideo App

**Figure 1:**
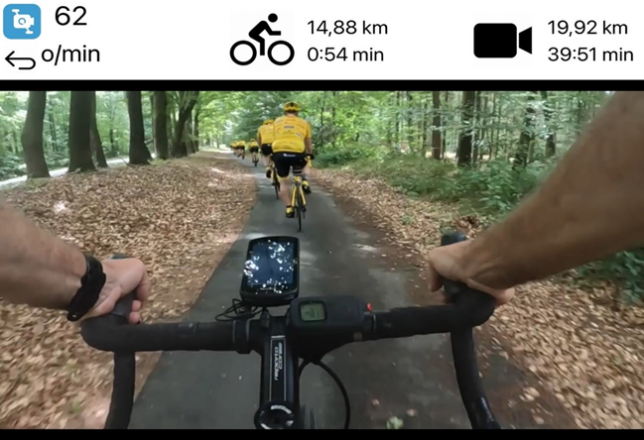
A screen capture of the video on the iPad while cycling. The icon with 62 o/m shows the number of revolutions per minute. The cycling icon shows the performed distance and time. The video icon shows the distance and time of the video. With permission from 4Mvideo.

For individuals with COPD who are unable to cycle outdoors due to health or environmental barriers, virtual solutions can provide a comparable experience from home. The 4Mvideo app is a software system that allows users to experience outdoor cycling virtually by using a cycle at home. The individuals can cycle along a recorded route featuring a cyclist, thus gaining a feeling of real cycling without the challenges of outdoor exercise. For this project, local cyclists will document their journey from Aalborg (Denmark) to Paris (France) with several hours of videos. This content is accessible through the 4Mvideo app, enabling users to virtually cycle from Aalborg to Paris from their homes (Figure 1).

**Figure 2:**
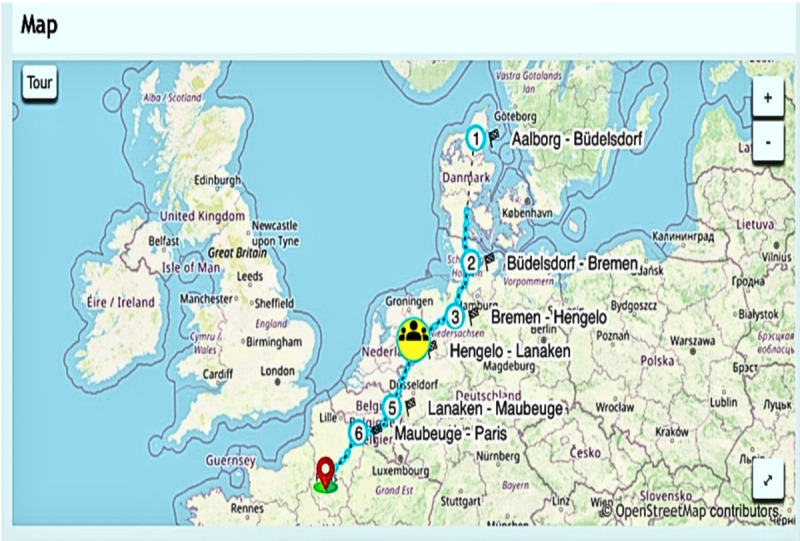
Map showing the team’s progress on the virtual tour to Paris. With permission from 4Mvideo.

Cyclists participated in teams, and the app had a chat feature to simulate the experience of cycling together as a team. The collective distance covered by the team each day is tracked and shown for the participants, allowing the group to virtually travel from Aalborg to Paris over a 7-week period, regardless of individual contributions. Resistance on the bicycle and the app's responsiveness to pedal rotations are customized according to each participant's exercise tolerance. A map feature helps the individuals track their progress and increases motivation by allowing the participants to visualize their journey (Figure 2). While the app addresses logistical and environmental barriers, it is equally important to consider the motivational aspects that encourage sustained participation in home-based exercise programs. SDT provides a framework for considering these motivational drivers.

### Self-Determination Theory

SDT offers a framework to understand motivational drivers [13]. SDT suggests that motivation is fostered by 3 basic psychological needs: *autonomy* (the sense of control over one’s action), *competence* (the ability to master tasks), and *relatedness* (feeling connected to others) [13]. When these needs are fulfilled, the individuals are more likely to experience intrinsic motivation, leading to greater engagement in sustained behaviors, such as exercise.

To analyze the results of a home-based cycling exercise intervention for individuals with COPD, SDT provides a valuable framework for understanding motivation and behavior change in home-based cycling exercise intervention for individuals with COPD. The study aims to explore the development of a home-based cycling exercise intervention for individuals with COPD, focusing on aspects such as bicycle selection, app functionality, and pilot testing. Furthermore, it aims to explore participants' and nonparticipants' attitudes toward the intervention.

## Methods

### Study Design

This qualitative study was based on a phenomenological-hermeneutic approach, which aimed to explore participants’ attitudes and experiences to inform the development of an intervention. This approach explored both the participants' lived experiences and the interpretation of these experiences [14]. It was conducted through individual semistructured telephone interviews. An inductive thematic analysis was used to analyze the data. This study was reported in line with the COREQ (Consolidated Criteria for Reporting Qualitative Research) guideline [15]. This qualitative study was a part of a noninferiority randomized controlled trial conducted at Aalborg University Hospital for the COPDtoParis project [16].

### Informants

Inclusion criteria were defined as persons living with COPD with experiences relevant to the development of the intervention. Recruitment was based on a purposive sampling, selecting informants who could provide diverse insights for the development of the intervention. The number of participants was determined based on information power, with recruitment concluding when the informants provided sufficient content to elucidate the research question [17]. The number of participants was evaluated based on whether each new informant contributed significant new information. Recruitment was concluded once thematic saturation had been reached and no substantial new themes or variations in depth were present in the data to address the study aim. Once sufficient variation and depth in the informants' responses had been reached to answer the research question, the data volume was considered adequate.

Groups selected to inform the development process were (1) test pilots, who were known from the research unit, had COPD, and were assessed by a doctor to be capable of handling home cycling; (2) participants in COPDtoParis; and (3) nonparticipants, individuals who were eligible but declined to participate in COPDtoParis. Additional characteristics of the informants were presented in Table 1.

**Table 1:** Informant characteristics.

Informant identification	Group	Age (years)	Sex
TP^a^ 1	Test pilot	61	Female
TP 2	Test pilot	69	Female
PP^b^ 3	Participated	71	Male
PP 4	Participated	75	Female
PP 5	Participated	72	Female
PP 6	Participated	76	Female
PP 7	Participated	78	Female
NP^c^ 8	Participated	71	Male
NP 9	Nonparticipants	79	Female
NP 10	Nonparticipants	66	Female
NP 11	Nonparticipants	65	Female
NP 12	Nonparticipants	66	Male
NP 13	Nonparticipants	72	Female
NP 14	Nonparticipants	77	Female
NP 15	Nonparticipants	72	Female

^a^TP: test pilot.

^b^PP: participant.

^c^NP: nonparticipant.

### Data Collection

The interview guide included open-ended questions, which allowed specific topics to be addressed while also leaving space to explore new subjects brought up by the informants. Individual semistructured telephone interviews were conducted with the test pilots, participants, and nonparticipants. Nonparticipants were asked “Why did you refrain from participating in the project?” and were encouraged to elaborate where relevant.

The interview guide was based on the SDT framework and covered topics related to the practical implications of applying a home-based cycle exercise for individuals with COPD (see Table 2).

**Table 2:** Interview guide.

Theme	Examples of content
Introduction	Details provided to participants about the interview process and its purpose.
Autonomy	“What motivated you to participate in the cycling project?” “What motivates you to exercise on the bicycle?” “What do you think about home exercise versus going out?”
Competence	“How is it to use the bicycle? Is it easy or difficult to use?” “What is your overall experience with the bicycle?”
Relatedness	“How do you experience the social aspect through the tablet?” “How do you feel you have been supported in your home exercising?” “What additional support do you believe you need to succeed with your home exercise bicycle in the future?”
Brings the interview to a close	“What do you feel was missing or could have been improved?”
Additional	Opportunity for participants to elaborate if they felt any important topics had been overlooked.

All interviews were conducted by a researcher who had an existing relationship with the participants through their involvement in the COPDtoParis study. The interview guide was pilot tested during an initial interview, which resulted in only minimal adjustments. Therefore, the pilot interview was included as part of the data. The interviews were audio-recorded and transcribed. They were conducted from August 2024 to September 2024.

### Data Analysis

The analysis followed Braun and Clarke’s 6 phases of thematic analysis [18]. Transcripts were read naively to ensure familiarity. Initial coding was generated inductively and then reviewed collaboratively. Themes were developed through discussions among the research team [18]. Saturation was considered achieved when no new themes emerged from 3 consecutive interviews. Reflexivity was ensured through regular team discussions, with explicit attention to the lead researcher's role as a known figure to participants. While no formal triangulation was conducted, interpretations were validated collaboratively.

### Ethical Considerations

The study was conducted in accordance with the ethical standards outlined in the Helsinki Declaration [19] and followed the data protection standards set by the Danish Data Protection Agency [20]. In Denmark, qualitative interview studies that do not include biological material do not require approval from a national ethics committee. Before participation, all informants were fully informed about the study’s aim and provided oral consent. All data were anonymized and securely stored in accordance with the General Data Protection Regulation [20]. Participants did not receive financial compensation.

## Results

The results reflected the perspectives of 15 informants, providing insights into their experiences with the home-based cycling exercise intervention. These experiences, derived from semistructured interviews, were analyzed into 7 themes that highlighted practical and emotional dimensions of the intervention, focusing on patterns and variances. Informants’ quotations were included to illustrate the results.

### Choosing the Bicycle: Stability Versus Portability

The selection and suitability of the bicycle was guided by the target group’s physical limitations. High-performance bicycles were deemed unsuitable due to issues with balance and mobility, leading to the choice of pedal bicycles. Three models were tested by test pilots, and feedback resulted in selecting a stable, compact model. On smooth floors, the bicycle scooted forward while pedaling, but placing it on a thin yoga mat prevented this. It remained stable on carpeted surfaces.

The following quote emphasized the importance of the bicycle’s practical design, including its physical size and space requirements:

I think the bicycle is practical because it doesn't take up as much space as a regular bicycle.

Participant 8

While participants appreciated the practicality and stability of the chosen bicycle, some found its weight problematic. For instance, one participant noted difficulty moving it:

I have a cleaning lady and I need to move it when she comes. I can't lift the bicycle myself because it’s so heavy. Therefore, I would rather use my own pedal bicycle, which I can lift.

Participant 6

Overall, the design was deemed suitable for the participants:

I think it’s wonderful because I can move it around as it suits me.

Participant 5

I think it’s super. It has been easy to figure out how to use the bike.

Participant 4

Preferences varied significantly, and the feedback underscored the delicate balance between the bicycle's weight, which ensured stability, and the challenge it posed for some participants due to its heaviness. This highlighted the diversity in individual needs, with some participants valuing the added stability, while others found the weight to be a barrier due to their health limitations. These variations demonstrated that no single bicycle could fully accommodate the needs of all participants.

### Geographical Awareness and Video Quality

The videos in the cycling app received mixed feedback. Some informants appreciated the landscape views, feeling motivated by familiar landmarks, while others preferred alternative distractions like television. The video content's quality and relevance were highlighted as factors influencing engagement. Issues such as impaired hearing or visual barriers also impacted the usability of the videos.

I can’t hear their talk because of my bad hearing. Therefore, I don’t really use the videos but cycle while I watch TV.

Test pilot 2

The lack of identifiable landmarks or city signs led to a sense of disorientation, making it difficult for some participants to stay motivated.

I like the parts near home. When they cycle south of my hometown, I don’t know where I am, and that’s less motivating.

Test pilot 1

Due to feedback from the test pilots, a feature providing an overview linked to a map was added to the app. In this section of the app, participants can view which routes are associated with specific geographical locations. Furthermore, this feature offers a clear overview of which routes have already been completed and which still need to be cycled.

A participant appreciated the videos featuring beautiful landscapes, expressing that they felt like a part of the cycling group in the videos:

I enjoy biking along with the videos, it's cozy, beautiful landscape and I feel like I'm part of the trip. Other times, I watch TV while doing it. It varies.

Participant 6

The participants enjoyed watching the landscape while cycling; however, the participants felt the filming quality could be improved:

I think it's fun to sit and look at the landscape when I'm cycling. However, I sometimes think there are too many cyclists in front of the one filming, and it blocks my view of the landscape.

Participant 4

The virtual landscape was perceived as valuable for enhancing motivation among the participants. While some participants expressed a wish for more visually appealing content, their focus on the journey and the landscape, rather than the specific goal of reaching Paris, implied that the journey itself was more engaging than the destination.

The videos were considered motivating by some participants, while others actively chose other activities, such as watching television while cycling. This suggested that the videos were a supplementary feature appreciated by some participants. It was not the presence of the videos but rather the content that was highlighted as important for engagement.

### Connecting Online: Knowing the Teammates

During the test pilot phase, the test pilots were asked if an online chat room could enhance their experience of cycling in terms of getting to know their teammates and improving motivation and adherence. The test pilots expressed a positive attitude toward the notion; therefore, the chat function was developed for the app.

All participants were asked if they wanted access to the chat function, which was intended to increase a sense of community and support. There were significant differences in what participants thought about the chat function. The commonality was that none of them had used the feature. Some participants expressed that they could not see the chat button because it was too small. Others emphasized that it was not a relevant function for them, as they did not want to chat with strangers:

To be honest, I don't think anyone will use it. Because you don't know the people and you don't know who they are.

Participant 4

Others stated that it might be interesting to try:

I haven't tried the chat function yet, maybe I will at some point. I haven't decided on that yet.

Participant 3

I might use the chat function at some point. I just need to start using the bicycle a little more. Anyway, I'm positive to try it out.

Participant 7

Based on the quotes, the chat function had not had the desired impact at the time. None of the participants reported using the chat feature, highlighting a disconnect between its intended purpose and participants’ needs. Some participants mentioned that they would have been more willing to use the chat if they had the chance to meet the other participants first, as they would no longer be strangers.

### Need for Individual Guidance

The participants showed variation in their capacity to manage intensity. Some required a lower intensity level with assistance, while others were able to self-manage effectively. One test pilot needed explicit guidance regarding the intensity of the exercise, as the test pilot reported difficulties in self-regulating. This led to negative physical consequences, such as muscle soreness. The muscle soreness caused concern for the test pilot, as illustrated by the following statement:

Today, my legs are very sore after cycling. I didn’t feel the strain on my legs while I was cycling, but the day after I was so sore and could hardly walk.

Test pilot 2

This highlighted the importance of providing clear guidelines to prevent overtraining, particularly for participants with poor health conditions like COPD. It was also important to inform participants that some leg discomfort was normal and harmless.

Participants valued the ability to adjust the intensity level, as their physical capabilities varied daily due to energy levels and health conditions. This was expressed in a statement:

Some days I have the energy for a higher intensity, while other days I have less. I adjust it myself as it suits my energy level and mood.

Participant 6

This statement illustrated the necessity for individual adaptation to the intensity level that accommodated the participants’ energy levels and moods. Explicit guidance was essential to help prevent overexertion and associated discomfort while allowing for individual adjustment based on daily variations in energy and capability. When providing tailored guidance on adjusting the intensity, no issues with intensity adjustment were reported by other participants.

### Flexibility as an Important Factor

Another important theme among the participants was the flexibility provided by the home-based exercise intervention. They valued the ability to cycle at different times throughout the day based on personal preference, which gave them a sense of control over their exercise routines. One participant expressed this point:

If I feel like it at 10 AM, then it's frustrating if I must wait until 2 PM to cycle. Now I can go and sit down no matter what time of day. I decide when I have the air and energy for it.

Participant 7

The flexibility allowed participants to exercise when they felt physically capable, thereby enhancing adherence to exercise. Participants described significant variability in their exercise capacities, reflecting the need for flexible scheduling and personalized approaches:

One day it's hopeless, the second day it's okay and the third day it's great.

Participant 3

This flexibility was crucial for maintaining motivation and adherence, particularly for those dealing with poor health conditions. Autonomy in choosing exercise timing and adaptability to health conditions were key factors that supported participating in the intervention.

### Barriers Related to Physical and Mental Strain

Energy levels and prioritization were significant factors influencing participation in the intervention. Nonparticipants described a range of barriers that were closely connected to their physical condition and mental state. For the participants, the most important challenge was severe symptoms such as exhaustion and shortness of breath, which made physical activity unmanageable. These symptoms often left them with little energy for exercise, as illustrated below:

I feel best when I'm lying down or sitting. If I'm physically active, I feel very unwell. I don't have the energy or strength to exercise...I'm constantly exhausted and could just sleep all the time.

Nonparticipant 9

Other nonparticipants chose not to prioritize exercise. For some, their priorities were everyday obligations like work, or they were already feeling sufficiently active through other activities. This was expressed in the following quote:

I feel that I’m already very active, so there’s no need for more. There isn’t much room for additional activity.

Nonparticipant 13

One nonparticipant had negative experiences with pedal bicycles and found cycling generally unattractive:

Cycling has never been for me, and I have had several of those pedal bicycle at home, and it has not been with any good results.

Nonparticipant 15

These accounts illustrate how nonparticipation was not only due to physical limitations but also reflected individual priorities and prior experiences with exercise, shaping attitudes toward exercise.

Beyond physical symptoms and practical considerations, nonparticipants described how stress and mental overload influenced their decisions. They expressed feeling overwhelmed by their health situation and the amount of health care offers they had to manage. This experience of overload created a mental barrier to participation, as described by a nonparticipant:

I have felt distressed ever since I was discharged from the hospital. I can't fit anymore into my home, everything is cluttered. I feel like I can't manage anymore.

Nonparticipant 14

The strain of balancing physical limitations, practical constraints, and mental distress made participation challenging for some. This ongoing stress since being discharged from the hospital has impacted their ability to manage additional responsibilities or activities, including the cycling intervention. These narratives from nonparticipants highlight how psychological and emotional strain enhanced practical barriers, making nonparticipation a coping strategy rather than a simple disengagement.

### The Home Environment as Both Facilitator and Barrier

Participants and nonparticipants described the home environment as central to their experience of the cycling intervention. For participants, exercising at home offered comfort, accessibility, and flexibility. The ability to place the pedal bicycle in familiar spaces, such as the living room or dining area, meant they could exercise while engaging in everyday activities like watching television or using a computer. One test pilot highlighted the privacy and psychological ease of exercising at home:

When it comes to exercising, I prefer to do it alone. That's when I get the most out of it. I feel best exercising by myself and in familiar surroundings.

Test pilot 1

A sense of privacy and autonomy enhanced motivation and made exercise feel more sustainable for participants. Similarly, other participants emphasized that exercise at home ensured regular engagement, since going outside or to a fitness center would have been too demanding. For these participants, home-based exercise was not only more appealing but also allowed them to adapt routines to their fluctuating condition:

The fact that the exercise takes place at home ensures that I actually engage in exercise. If I had to go to a fitness center, I would never attend. By the time I arrived, I would already be exhausted.

Participant 5

In contrast, lack of space was a repeated barrier for nonparticipants. They described their homes as overcrowded with furniture and assistive devices such as walkers and oxygen flasks. Living in smaller apartments, combined with many appointments and care persons coming and going, led nonparticipants to express that they lacked space for more items or engagements. One nonparticipant stated:

I live in a 60 square meter apartment, and I already have an oxygen machine and a lot of flowers, so I simply don’t have room for it. I know I could just put it aside, but I honestly don’t have the energy to keep anything else here. I have just started receiving home care for cleaning, and I must move everything of the floor. I simply can't manage it anymore.

Nonparticipant 10

The nonparticipants’ struggles with limited space highlighted the importance of considering the practical realities of living environments when introducing home-based interventions. For individuals already managing a high degree of clutter and aids, even small additional equipment felt overwhelming. These physical constraints often interacted with feelings of fatigue and stress, making participation in exercise interventions less feasible.

In contrast, participants found the bicycle compact and movable, which is why they did not report concerns about space and practical limitations. The differing experiences between participants and nonparticipants suggested that perceived or actual lack of space was not only a physical constraint but also a psychological barrier. For nonparticipants, the anticipation of managing additional equipment might have amplified feelings of being overwhelmed, even if the equipment itself was designed to be compact. This contrast shows how the home environment could either enable or obstruct exercise, depending on individual circumstances, thereby underscoring the contextual nature of participation.

## Discussion

### Summary of Key Findings

The development of a home-based cycling exercise for individuals with COPD aimed to overcome challenges associated with center-based exercise, which often presented logistical barriers, such as transportation and mobility issues. These barriers were particularly relevant for individuals with advanced COPD, who frequently experienced reduced mobility or required supplemental oxygen. Home-based exercise provided the advantage of enabling participation in familiar surroundings, aligning with previous findings that highlighted the benefits of remaining at home while participating in digital health interventions to promote comfort and autonomy [21].

### The Journey Was More Engaging Than the Destination

The development and design of virtual maps, incorporating clear markers or familiar landmarks, aimed to enhance participants’ motivation. However, the study results revealed that the journey itself was often more engaging for participants than achieving specific goals. This underlined the importance of improving the visual quality of videos by optimizing perspectives and reducing distractions. Beautiful surroundings and engaging visuals motivated participants, suggesting that future interventions should address the preferences of both those motivated by visual content and those who were goal oriented. In practice, this could involve offering a library of cycling routes with different themes (eg, familiar local areas, coastal landscapes, or urban routes), allowing participants to select according to personal preferences. Furthermore, technical improvements such as higher resolution filming and minimizing visual obstructions (eg, cyclists blocking the view) may increase immersion. To ensure scalability, such features could be integrated into existing digital health platforms, with physiotherapists or municipal health services providing initial guidance on setup and personalization. These concrete adjustments may help transform generic suggestions into actionable design elements that strengthen engagement and long-term adherence.

### The Bicycles’ Design

The participants expressed satisfaction with the bicycle's design and placement, but some encountered challenges due to the weight and portability of the bicycle. For individuals with COPD, who often experience reduced physical strength and mobility, the ability to easily relocate the bicycle within their home is important. It might be necessary in the future to offer different types of bicycles, a lightweight and a heavyweight, or provide guidance on creating a permanent and accessible space for the bicycle. This would enhance autonomy, a key element in SDT, by ensuring that participants feel in control of their exercise environment.

### Autonomy and Competence—The Perspective of SDT

From the perspective of SDT, *autonomy* and *competence* were identified as particularly important motivational factors. *Autonomy* was facilitated by the flexibility to exercise when participants felt physically capable. Participants noted that the availability of the equipment at home was crucial for maintaining motivation. *Competence* was fostered through adjustable cycling intensity, which enabled participants to experience progress and mastery. Similarly, participants felt a sense of competence when they were able to plan their own exercise sessions. These results were consistent with prior research, which reported increased adherence to self-managed exercise programs among older individuals who felt competent [22].

Although autonomy and competence emerged as central motivational drivers in this study, the role of relatedness appeared less pronounced. Participants showed limited interest in using the chat function and emphasized privacy rather than social connectedness during exercise. This finding contrasts with the literature in digital health, where social support is often identified as a key facilitator of engagement and adherence to physical activity programs [23,24]. An explanation for this discrepancy may be the specific context of individuals living with COPD, many of whom experience fluctuating health, fatigue, and a strong need to conserve energy. For these individuals, the opportunity to exercise privately and on their own terms may outweigh the potential benefits of social interaction. Another explanation could be the design of the intervention itself. Participants did not know each other prior to the study, and the virtual chat function may not have provided a sufficiently meaningful basis for social connectedness.

These findings indicate that relatedness should not be dismissed as unimportant, but rather reconsidered in terms of how it can be meaningfully facilitated for this specific population. Future interventions could explore hybrid approaches, such as introductory group meetings or small-group video sessions, to establish a sense of familiarity and trust before transitioning to digital platforms. At the same time, it is important to acknowledge that some individuals may prefer solitude and autonomy, highlighting the need for flexible designs that can accommodate varying preferences for social engagement. By situating our findings within the broader literature, it becomes evident that relatedness remains an important motivational factor, but its relevance and manifestation may depend strongly on the population, health status, and context in which the intervention is delivered.

### Barriers From the Nonparticipants

Although exercise is widely known to be beneficial, this intervention was not suitable for everyone. Nonparticipants identified barriers such as lack of energy, stress, mental strain, and practical issues like space constraints. One nonparticipant’s negative experiences with pedal bicycles highlighted the importance of accommodating personal preferences in exercise design.

Individual histories and past experiences also played a role in engagement. For example, a participant who expressed enthusiasm for the idea of cycling to Paris may have had prior experiences with group cycling or goal-oriented activities, which positively influenced motivation. In contrast, those less focused on collaborative or goal-driven aspects may not have shared the same background or attachment.

Nonparticipants express feeling overwhelmed by the multitude of health care offers and the overall demands of their condition. This indicated that the mental strain of managing health and daily life responsibilities often overshadowed the potential benefits of engaging in the home-based cycling exercise intervention. Living in smaller or cluttered spaces expressed frustration with the lack of physical room to accommodate the equipment.

To address these issues, interventions should consider strategies to reduce mental overload, such as simplifying health care offers and minimizing the introduction of additional equipment or activities into already crowded and stressful environments. Practical solutions, such as compact or foldable exercise equipment, could also mitigate space-related challenges.

The responses from nonparticipants indicate that this intervention could be most suited for a certain group of individuals with COPD: those who are significantly impacted by their condition, largely homebound, but not dependent on personal care or disability aids. It did not appeal as much to those still capable of and motivated by engaging in activities and exercise outside their home.

Our findings are consistent with earlier work showing that individuals with COPD often prefer home-based exercise because it offers autonomy, privacy, and flexibility in order to be engaging [25,26]. The barriers and motivators we found in our study were further reflected in a systematic review by Robinson et al [27]. They identified key subthemes affecting engagement, including self-efficacy, feedback, support, peer interaction, and creating routines. Likewise, they found common barriers such as exhaustion, space constraints, and emotional strain, which were also reflected in a study [27,28].

Further studies are needed to identify exercise options that could be motivating and acceptable for the nonparticipants, who declined from participating due to resource limitations. While well-established exercise programs already exist for the more active individuals, in line with the points made by Wouters et al [9], there is a need to develop and investigate tailored exercise offers for less mobile patients. COPDtoParis could provide a relevant low-barrier exercise alternative for homebound patients with COPD motivated by home-based options. Tailoring the intervention further to this group could help fill a gap in existing offers, addressing both physical limitations and practical constraints.

### Strengths and Limitations

#### Strengths

A strength of this study was the inclusion of both participants and nonparticipants, which allowed for a more comprehensive understanding of the barriers and facilitators to engagement in the home-based cycling exercise intervention. By capturing insights from both groups, the study not only highlighted the experiences of those who engaged in the intervention but also the reasons for nonparticipation, offering a more nuanced understanding of the intervention’s reach and potential areas for improvement. Another strength was the theoretical foundation of SDT for both the interviews and the analysis. This theoretical aspect ensured that the results were not only descriptive but also linked to a broader psychological understanding. The thematic analysis by Braun and Clarke's [18] method strengthened the study’s rigor. By following a structured, step-by-step approach to identify, analyze, and report themes, the analysis remained transparent and systematic. One researcher conducted the analytical process, ensuring consistency in coding and theme development, while the other researchers validated these results through discussions. Furthermore, the analysis was presented transparently and systematically, with quotes representing nearly all the informants, which enhanced the trustworthiness of the analysis and results.

#### Limitations

As the interviewer was familiar with the participants, reflexivity was emphasized throughout the process. An audit trail documenting coding decisions was maintained, and coding structures were discussed within the research team to ensure transparency and minimize bias.

Although the inclusion of nonparticipants is a strength of this study, the depth of their accounts was more limited compared to participants’ interviews. Contact with nonparticipants was less extensive, and data were collected through telephone interviews. This restricted the degree of thematic interpretation.

While SDT provided a useful motivational lens, relying solely on this framework may have limited our analysis. Other models, such as the COM-B (Capability, Opportunity, and Motivation for Behavior Change) model, could have provided a more comprehensive behavioral systems approach, particularly in relation to environmental and capability-related barriers. Similarly, the UTAUT (Unified Theory of Acceptance and Use of Technology) framework might have offered insights into participants’ adoption of the app and its technological usability. Future research could benefit from combining SDT with complementary frameworks to capture both motivational and structural determinants of engagement.

### Conclusions

This study highlights the value of home-based cycling for individuals with COPD, particularly the importance of autonomy, flexibility, and tailored guidance. For clinicians, the findings suggest offering clear instructions on intensity adjustment and considering patients’ daily symptom fluctuations. For developers, practical improvements include customizable training schedules and enhanced video quality with diverse route options. Barriers such as limited space and health care overload underline the need to assess home environments and current treatment burden before enrollment, which may inform both exclusion criteria and supportive services. Future research should evaluate the intervention in randomized controlled trials, cost-effectiveness, and tailoring based on SDT profiles.

## Abbreviations

COM-B: Capability, Opportunity, and Motivation for Behavior Change
COPD: chronic obstructive pulmonary disease
COREQ: Consolidated Criteria for Reporting Qualitative Research
PR: pulmonary rehabilitation
SDT: self-determination theory
UTAUT: Unified Theory of Acceptance and Use of Technology
